# Hepatitis C in the criminal justice system: opportunities for global action in the era of viral hepatitis elimination

**DOI:** 10.1186/s12916-020-01680-0

**Published:** 2020-08-14

**Authors:** Matthew J. Akiyama

**Affiliations:** Albert Einstein College of Medicine/Montefiore Medical Center, 3300 Kossuth Ave, Bronx, NY 10467 USA

**Keywords:** HCV, Prison, Jail, Criminal justice system, Correctional setting, Substance use disorders, PWID, HCV elimination, DAA

## Background

In recent years, highly effective, well-tolerated, all oral direct-acting antiviral (DAA) therapies for hepatitis C virus (HCV) have raised the prospect of an era in which HCV elimination is achievable worldwide. Globally, 71 million individuals are estimated to be living with HCV. In 2016, the World Health Organization (WHO) issued viral hepatitis elimination targets that include diagnosing 90% of individuals living with HCV, initiating 80% of those who are eligible on treatment, and reducing incidence by 90%. While the WHO’s goals have been made more attainable due to the availability of these new effective therapies, recent modeling data suggest that 80% of high-income nations are not on track to achieve them by 2030 and nearly 70% are not expected to meet them before 2050 [1].

Globally, HCV disproportionately affects individuals who intersect with the criminal justice system. Of the estimated 10.2 million people incarcerated worldwide on any given day, approximately 15%, or 1.5 million, are living with HCV [2]. Despite the dramatic epidemiologic disparity between corrections and the community, HCV is overwhelmingly transmitted outside correctional settings. HCV is far more common among marginalized populations such as people who use drugs, the homeless, and mentally ill. These groups are overrepresented in correctional settings due to policies related to the criminalization of drug use and crimes of poverty. High HCV rates coupled with a point of contact with these marginalized populations make correctional settings critically important to provide all phases of the HCV care cascade (screening, linkage to care, treatment, and prevention).

## The HCV care cascade in correctional settings: missed opportunities and priorities for action

As the first steps in the care cascade, screening and confirmation of chronic HCV infection are important to ensure individuals are aware of their HCV status. Not only is HCV screening in correctional settings complicated by a lack of HCV-related knowledge, incarcerated individuals often do not provide a complete history of injection drug use due to the stigma associated with substance use disorders and fear of incrimination [3]. Therefore, risk-based, targeted screening may miss opportunities to make HCV diagnoses. Universal opt-out HCV testing upon entrance in correctional settings can help mitigate stigma by routinizing testing and raising awareness about HCV infection [4]. In the USA, practice guidelines recommend routine opt-out HCV screening among all incarcerated individuals [5], yet, the majority are not doing so [6].

For individuals who are diagnosed, linkage to HCV treatment can be challenging. In general, correctional settings have even less access to DAAs than surrounding communities, particularly in low- and middle-income countries where global access is already poor. Even in nations with more widespread access to DAAs like the USA, the majority of facilities do not provide HCV treatment despite comparable outcomes in correctional settings [6, 7]. Moreover, the average length of stay for the one third of individuals residing in short-term US jail facilities is less than two weeks. Therefore, ensuring the completion of an 8- to 12-week course of DAAs is challenging. Similar challenges exist internationally for those on remand. Despite the quick turnover and inability to administer and track the full course of DAAs, short-term correctional settings serve as an opportunity to continue HCV-care for those already on treatment but also have the potential to engage a large number of individuals unaware of their status into care, and provide testing and education for those at risk for HCV. A study conducted in the New York City jail system found viral load suppression for over 90% of patients including those who initiated treatment prior to incarceration and those who initiated treatment in the jails, demonstrating the feasibility of DAA access in short-term settings [8].

Upon release, justice-involved individuals face barriers related to social and structural determinants of health such as poor social support and housing, as well as relapse to active substance use. This makes it imperative to connect individuals to community-based social, behavioral, and addiction services prior to their release. One strategy is transitional care coordination, a patient navigation strategy that has proven successful to facilitate linkage to care for patients living with HIV [9]. Implemented on its own or in tandem with existing HIV-focused program, HCV-focused transitional care coordination can also improve linkage to and retention in HCV care [10]. Such programs offer advocacy, health education, and social support to people living with HCV who are incarcerated and can work with them to create a plan for initiation or continuation of HCV treatment upon return to the community.

Beyond justice-involved individuals themselves, the high rate of substance use disorders within this population indicates that this group plays a large role in community transmission. Therefore, HCV treatment in correctional settings not only benefits this high-risk population, but paves the way for preventing HCV transmission in the communities to which justice-involved individuals return. Correctional facilities can also provide HCV-related risk-reduction education to prevent new infections, which will not only lead to benefit for HCV, but should also be leveraged to prevent other blood borne and injection-related infections. Linkage to opioid agonist therapy and resources for syringe exchange will also be invaluable to these efforts.

## Leveraging correctional settings to support HCV elimination: a call to action

As HCV continues to disproportionately affect individuals involved with the criminal justice system, the development of effective strategies to promote testing, diagnosis, and linkage to care during incarceration is an opportunity to improve the coordination of care among this high-risk population. Strengthening the HCV-care cascade in correctional settings will move us closer achieving international reduction in new HCV infections, improving health outcomes for people living with HCV, and reducing HCV-related health disparities. To make HCV elimination conceivable in the coming decades, we must amplify international political will to tackle HCV in the criminal justice system; without action among justice-involved individuals, achieving the WHO elimination goals will be difficult to attain.

## Data Availability

Not applicable.
